# High diversity and no significant selection signal of human *ADH1B* gene in Tibet

**DOI:** 10.1186/2041-2223-3-23

**Published:** 2012-11-23

**Authors:** Yan Lu, Longli Kang, Kang Hu, Chuanchao Wang, Xiaoji Sun, Feng Chen, Judith R Kidd, Kenneth K Kidd, Hui Li

**Affiliations:** 1 State Key Laboratory of Genetic Engineering and MOE Key Laboratory of Contemporary Anthropology, School of Life Sciences, Fudan University, Shanghai, China; 2 Tibet University for Nationalities, Xianyang, Shaanxi, China; 3 Department of Genetics, School of Medicine, Yale University, New Haven, CT, USA

## Abstract

**Background:**

*ADH1B* is one of the most studied human genes with many polymorphic sites. One of the single nucleotide polymorphism (SNP), rs1229984, coding for the Arg48His substitution, have been associated with many serious diseases including alcoholism and cancers of the digestive system. The derived allele, ADH1B*48His, reaches high frequency only in East Asia and Southwest Asia, and is highly associated with agriculture. Micro-evolutionary study has defined seven haplogroups for *ADH1B* based on seven SNPs encompassing the gene. Three of those haplogroups, H5, H6, and H7, contain the ADH1B*48His allele. H5 occurs in Southwest Asia and the other two are found in East Asia. H7 is derived from H6 by the derived allele of rs3811801. The H7 haplotype has been shown to have undergone significant positive selection in Han Chinese, Hmong, Koreans, Japanese, Khazak, Mongols, and so on.

**Methods:**

In the present study, we tested whether Tibetans also showed evidence for selection by typing 23 SNPs in the region covering the *ADH1B* gene in 1,175 individuals from 12 Tibetan populations representing all districts of the Tibet Autonomous Region. Multiple statistics were estimated to examine the gene diversities and positive selection signals among the Tibetans and other populations in East Asia.

**Results:**

The larger Tibetan populations (Qamdo, Lhasa, Nagqu, Nyingchi, Shannan, and Shigatse) comprised mostly farmers, have around 12% of H7, and 2% of H6. The smaller populations, living on hunting or recently switched to farming, have lower H7 frequencies (Tingri 9%, Gongbo 8%, Monba and Sherpa 6%). Luoba (2%) and Deng (0%) have even lower frequencies. Long-range haplotype analyses revealed very weak signals of positive selection for H7 among Tibetans. Interestingly, the haplotype diversity of H7 is higher in Tibetans than in any other populations studied, indicating a longer diversification history for that haplogroup in Tibetans. Network analysis on the long-range haplotypes revealed that H7 in the Han Chinese did not come from the Tibetans but from a common ancestor of the two populations.

**Conclusions:**

We argue that H7 of *ADH1B* originated in the ancestors of Sino-Tibetan populations and flowed to Tibetans very early. However, as Tibetans depend less on crops, and therefore were not significantly affected by selection. Thus, H7 has not risen to a high frequency, whereas the diversity of the haplogroup has accumulated to a very high level.

## Background

The alchohol dehydrogenase (*ADH*) gene family has seven members expressed in different organs and tissues; *ADH1B* is expressed mostly in the liver and lungs [1], where most alchohol is dehydrogenated [2]. Therefore, *ADH1B* can be considered the most important member of the *ADH* family, and has become one of the most studied model genes for natural selection [3,4] among the human genes. A single nucleotide polymorphism (SNP), *ADH1B* Arg48His (rs1229984), results in large functional differences in the respective enzymes of the ancestral and derived alleles. The enzyme catalytic activity of the derived allele is 40 times that of the ancestral allele [5]. Thus, this SNP has been found to be relevant to cancers of the digestive and respiratory systems, alcoholism, addiction, and many other disorders [6-10].

The allele frequency of ADH1B*48His varies greatly among the world’s populations. The derived allele reaches high frequency only in eastern East Asia and Southwest Asia and is almost absent in the rest of the world [3]. Further study revealed that several SNPs in *ADH1B* form different haplotypes, and haplotypes with the ADH1B*48His allele are different in East Asia and Southwest Asia [4], with evidence in East Asia of strong positive natural selection [11,12] during the history of agriculture [13]. In western East Asia, the allele frequency of ADH1B*48His is not high [12], especially among Tibetans [4]. Tibetans share very recent common ancestors with the Han Chinese. Important questions are whether the ADH1B*48His alleles of Tibetans and Han Chinese have a common origin and, if so, why the frequency of this allele did not rise in Tibetans as it did in Han Chinese.

Our previous study found seven haplogroups (H1 to H7) for *ADH1B* among the world populations [14] based on seven SNPs in the gene. The ADH1B*48His allele appears in H5, H6, and H7. H5 is a Southwest Asian haplogroup. H6 derived from a crossover involving H5 and occurs primarily in East Asia and the Pacific region. H7 is derived from H6 by the addition of the derived allele of rs3811801 in the regulatory region of *ADH1B*. The age of H6 is about 15,000 to 21,000years [14], which is about the age of the modern East Asians [15-17]. Expansion of H7 happened only about 2,800 years ago, and is the only haplogroup with a strong signal of selection [14]. The frequency of H7 is much higher in Han Chinese than the frequency of H6 [12,18]. No study has previously investigated the distribution of the *ADH1B*haplogroups in Tibetan populations.

The languages of the Tibetans and Han Chinese belong to the Sino-Tibetan linguistic family. DNA evidence generally supports the hypothesis that populations speaking similar languages have recent common ancestors, especially in East Asia [19-21]. Y-chromosome DNA analyses argue that the divergence of Han Chinese and Tibeto-Burman populations was no earlier than 6,000 years ago [20,22,23].

There are five populations living in Tibet: Tibetans; Sherpa;Monba;Lhoba; and Deng [24]. The Tibetans are the major population of Tibet, divided into three major branches, Weizang in central Tibet, Amdo in the north, and Khams in the east. Two minor Tibetan populations, Tingri and Gongbo, are yet to be classified into the three branches. Monba, Lhoba, and Deng are all in southeast Tibet. Their languages are mostly in the North Assam branch of Tibeto-Burman, while three-quarters of the Monba people use dialects mixed with the Tibetan language. The Sherpa people live in the middle of the Himalayas, an area overlapping with China, Nepal, Sikkim, and Bhutan, and speak a language very close to Tibetan [25]. In this paper, we investigated Class I *ADH* and *ADH7*haplogroup diversity among all populations in Tibet, and examined the diversity and selection signal of *ADH1B*.

## Methods

### Population samples

**Figure 1:**
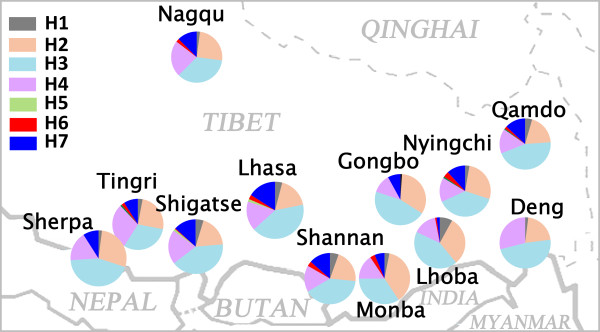
**Distribution of the population samples and *ADH1B *haplogroup frequencies.**

We collected blood samples of 1,175 individual samples from Tibet, including 1,009 Tibetans (Qamdo 157, Lhasa 334, Nagqu 24, Nyingchi 55, Shannan 147, Shigatse 192, Gongbo 50, and Tingri 50), 50 Lhoba, 16 Monba, 50 Deng, and 50 Sherpa. This sampling covers all regions of Tibet (Figure 1). All subjects were healthy and unrelated, and gave signed informed consent. Our research was approved by the Ethics Committee of Fudan School of Life Sciences.

### Experiments

DNA was extracted from the blood samples using Genomic DNA MiniPreps Kit for Blood (Generay Biotech Co., Shanghai, China). We examined 23 SNPs covering 173.5 kb ofthe *ADH* region (Additional file 1: Table S1), extending from rs1154473 upstream (telomeric) of *ADH7* through *ADH1C*, *ADH1B* (including ADH1B*48His), and *ADH1A* to rs1230025 downstream (centromeric) of the *ADH1* cluster. Seven of these covered the 16 kb of *ADH1B* and define the seven haplogroups (Additional file 1: Table S1). Taqman assays (Appliedbiosystems, Carlsbad, CA, USA) were employed for genotyping of the SNPs [26].

### Statistics

Allele frequencies of two core SNPs, rs1229984 and rs3811801, were estimated from the genotypes by simple gene counting assuming co-dominant inheritance and absence of null alleles. Geographic distributions of allele frequencies of these two SNPs in East Asia were transformed into contour maps using Surfer 8.0 (Golden Software, Golden, CO, USA). Both our results and data from literature were included [11,12,14,27-35] in the maps; we used the Kriging method for data interpolation.

Haplotypes of the 23 SNPs were determined using PHASE2.1 [36,37]. To make the haplotype estimation more reliable, our previous data of 4,362 chromosomes from 47 populations from the other region of the world were included as references [14]. *ADH1B*haplogroups were then determined according to the definitions of H1 to H7 [14].

Average gene diversity within haplogroups of each population was calculated using Arlequin 3.01 [38]. For haplogroups H5, H6, and H7, the relationships among the haplotypes of 23 SNPs were displayed by networks using Network 4.5.1.6 [39].

High extended linkage disequilibrium among the SNPs in the relevant genomic region might be signal of selection [40]. We used the long-range haplotype (LRH) test to examine the linkage disequilibrium and potential positive selection on the core haplotypes (rs1229984-rs6810842-rs3811801) of *ADH1B*[41]. Both extended haplotype homozygosity (EHH) and relative EHH (REHH) [42-44] were calculated in LRH tests. The integrated haplotype score (iHS) test was also employed to test for positive selection [44].

Principal component analysis was employed to assess the population relationships within the gene region. To identify the genetic barriers among the populations, pair-wise Fst values were estimated and the Barrier 2.2 software [45] was used.

## Results

### Haplogroup frequencies and diversities

We estimated 671 different haplotypes considering all 23 SNPs in all individual Tibetan samples (Table S1). These haplotypes were classified into 13 haplogroups for *ADH1B* (Table 1). There are three haplogroups containing the ADH1B*48His allele, H5, H6, and H7. H5 is very rare in Tibet. The frequency of H6 among the major Tibetan populations is only around 2%, and is absent in some minor populations. The frequency of H7 is higher than that of H6 in all populations, reaching around 12% in major Tibetan populations, and lower in the minor populations. ADH1B*48His is totally absent in Deng. A new haplogroup, H7b, was defined with the ancestral allele of rs1229984 and derived allele of rs3811801. This new haplogroup is almost absent outside of Tibet.

**Table 1:** **Frequencies of*ADH1B*haplogroups in Tibet**

	**Population**	**2N**	**H1**	**H1b**	**H1c**	**H2**	**H3**	**H3c**	H3*4	**H4**	**H5**	**H5b**	**H6**	**H7**	**H7b**
Major Tibetan	Qamdo	314	0.003	0.041		0.194	0.427		0.025	0.156	0.003	0.003	0.016	0.118	0.013
	Lhasa	668	0.001	0.039		0.178	0.409		0.006	0.166		0.015	0.025	0.138	0.022
	Nagqu	48		0.021		0.250	0.354			0.229			0.021	0.125	
	Nyingchi	110		0.027		0.273	0.364		0.018	0.155		0.009	0.036	0.118	
	Shannan	294	0.007	0.048		0.211	0.401			0.167			0.031	0.133	0.003
	Shigatse	384		0.044	0.003	0.190	0.406		0.003	0.203	0.005	0.008	0.005	0.120	0.013
Minor Tibetan	Tingri	100		0.030		0.250	0.300		0.010	0.290		0.010	0.020	0.090	
	Gongbo	100		0.010		0.330	0.450		0.010	0.120				0.080	
	Sherpa	100		0.020		0.280	0.430	0.010		0.170				0.060	0.030
	Monba	32		0.031		0.375	0.344			0.156			0.031	0.063	
Minority	Lhoba	100		0.080		0.310	0.430			0.150			0.010	0.020	
	Deng	100	0.010		0.010	0.210	0.480			0.290					

**Figure 2:**
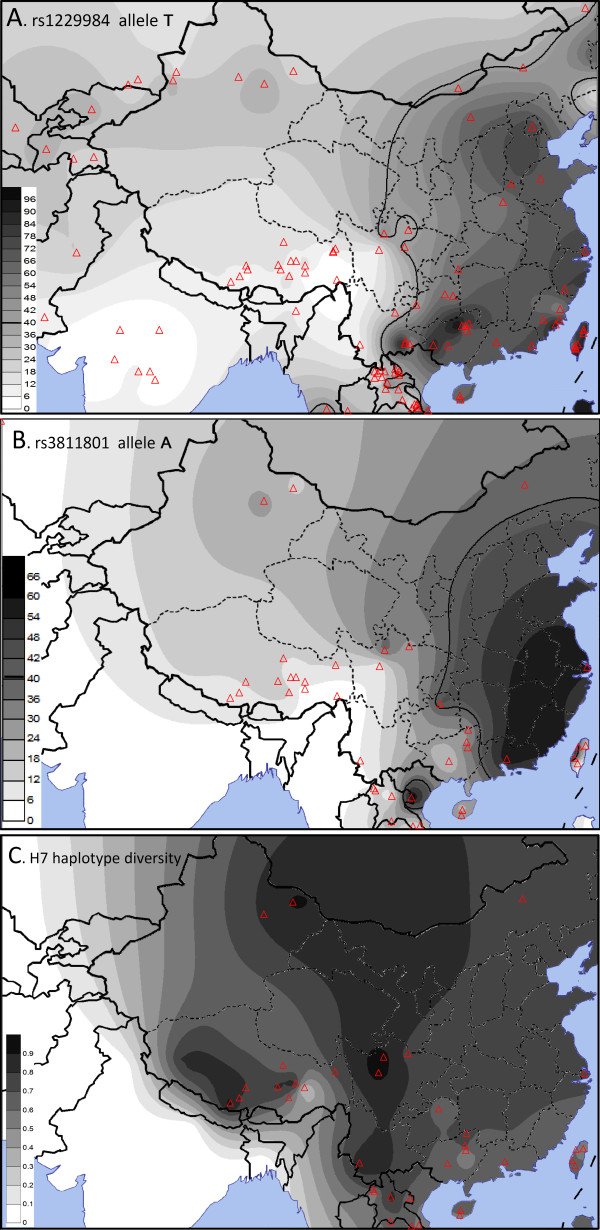
Geographic distributions ofADH1B*48His(rs1229984*T) and rs3811801*A and*ADH1B*H7 haplotype diversity in eastern Asia.

To assess the geographic distributions of ADH1B*48His (rs1229984*T) and rs3811801*A in western East Asia, we transformed the allele frequencies into contour maps (Figure 2A and B). The frequency of ADH1B*48His shows a clear decrease from east to west (Pearson correlation between longitude and the frequencies of rs1229984*T: r = 0.617, *P* = 2.27 × 10^-5^). The decrease to the west is smoother in the north than in the south. In Tibet, the frequency decreases slightly from north to south. This might indicate a migration of the Tibetans from north to south. Distribution of rs3811801*A is similar to that of ADH1B*48His but at lower frequencies (Pearson correlation between longitude and the frequencies of rs3811801*T: r = 0.673, p = 1.04 × 10^-15^).

Gene diversity was estimated from the haplotypes of 23 SNPs among haplogroup H7 and then transformed into a contour map (Figure 2C). Although H7 does not reach high frequency in Tibet, the within haplogroup diversity is very high. We also estimated the gene diversity of H7 within each linguistic family (Table 2). The total diversity of the Tibetans is the highest among all families.

**Table 2:** **Diversities (23 SNPs) of*ADH1B*haplogroup H7 among linguistic families**

	**2 N**	**Within haplogroup diversity**	**Average nucleic diversity**
Tibetan	2308	0.809 ± 0.016	0.116 ± 0.071
Hmong	240	0.748 ± 0.030	0.106 ± 0.067
Han	402	0.737 ± 0.026	0.084 ± 0.056
Tai-Kadai	950	0.732 ± 0.024	0.087 ± 0.057
Qiang	164	0.725 ± 0.066	0.122 ± 0.075
Altaic	646	0.724 ± 0.023	0.084 ± 0.055
Austronesian	342	0.657 ± 0.063	0.055 ± 0.041
Mon-Khmer	652	0.481 ± 0.063	0.079 ± 0.053

### Long-range haplotype networks

**Figure 3:**
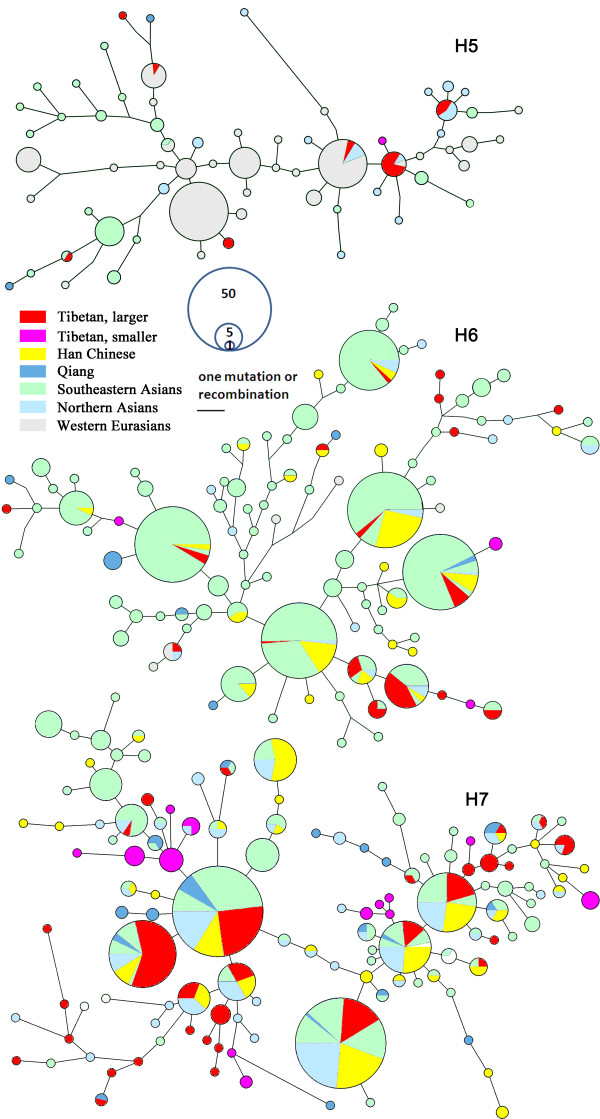
**Long-range haplotype networks of *ADH1B.*** We examined 23 SNPs covering 173.5 kb of the *ADH* region, extending from rs1154473 upstream (telomeric) of *ADH7* through *ADH1C*, *ADH1B* (including ADH1B*48His), and *ADH1A* to rs1230025 downstream (centromeric) of the *ADH1* cluster. For haplogroups H5, H6, and H7 the relationships among the haplotypes of 23 SNPs were displayed by networks using Network 4.5.1.6. The ‘Tibetan larger’ included 909 individuals from central Tibet (Qamdo 157, Lhasa 334, Nagqu 24, Nyingchi 55, Shannan 147, Shigatse 192), while the ‘Tibetan smaller’ included 216 individuals distributed in other parts of Tibet (Gongbo 50, Tingri 50, Lhoba 50, Monba 16, Deng 50, and Sherpa 50). The reference samples are all the population data from reference [12].

Similarities among the haplotypes can predict population relationships. We performed network analyses to assess the haplotype similarities (Figure 3). Haplogroup H5 is a West Asian type [14]. It is almost absent in East Asia, but appears in low frequency in Southeastern Asia and Northern Asia. We also found some H5 chromosomes in Tibet. According to the network, haplotypes of these samples were closely related to the Northern Asians, possibly indicating gene flow from Northern Asia to Tibet or from a common ancestor into both regions.

In the East Asian haplogroup H6 network, the major Tibetan populations share haplotypes with Southeastern Asians and Han Chinese, while the smaller populations have unique haplotypes, indicating distinct histories and possibly different origins of the populations in Tibet. This phenomenon also appears in the H7 network. The major Tibetan populations share some common haplotypes with other populations, while the minor populations have unique haplotypes. In the H7 network, the major Tibetan populations also have some unique haplotypes different from the haplotypes unique to Han Chinese. Therefore, we conclude that H7 haplotypes experienced different recent histories in the Tibetans and Han Chinese. The H7 haplotypes in Tibetans did not originate from Han Chinese but from the common ancestral population of Sino-Tibetan people.

### Positive selection test

**Figure 4:**
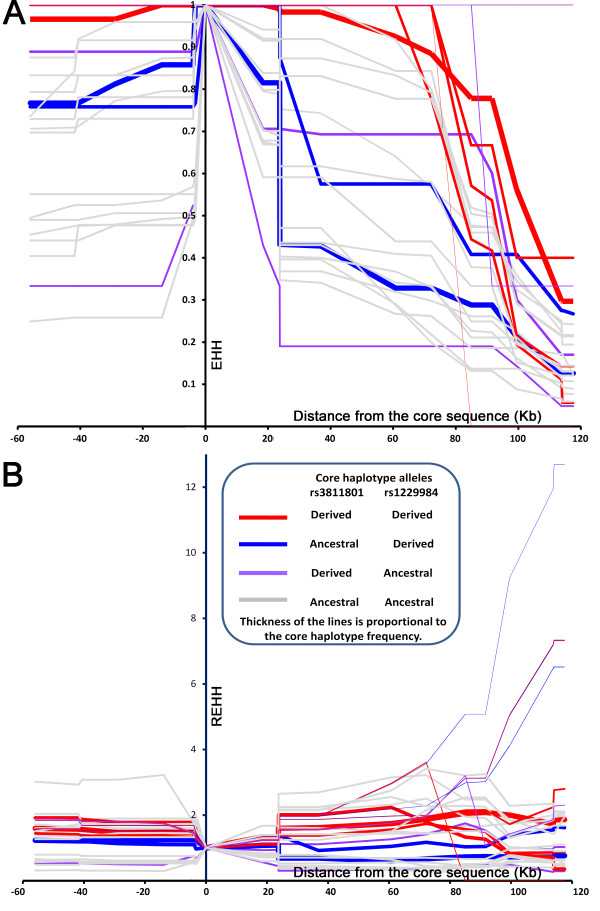
**Positive selection test (EHH and REHH) on *ADH1B *among populations in Tibet.**

The frequency of the youngest haplogroup H7 in Tibet is much higher than that of H6, suggesting positive selection might have increased the frequency of H7. We performed LRH analyses on the *ADH1B* gene to test for selection signals among populations in Tibet. LRH analysis has good performance for positive selection on low frequency alleles (approximately 10%) [46]. We calculated both EHH and REHH values of the core haplotype rs1229984-rs6810842-rs3811801 (Figure 4). EHH values of most haplotypes decreased rapidly from the core haplotype except for that of the haplotype with both derived alleles of rs1229984 and rs3811801 (H7). However, REHH did not show significant signals of selection for any haplotypes.

In addition, we performed the iHS test, which integrates and makes comparison between integrated extended haplotype homozygosity (iHH) of the ancestral allele and iHH of the derived allele for each SNP we examined. However, no signals of positive selection were observed (Additional file 2: Figure S1).

Therefore, we conclude that *ADH1B* H7 in Tibet has undergone only very weak, if any, positive selection.

### Population relationships for *ADH1B* region

**Figure 5:**
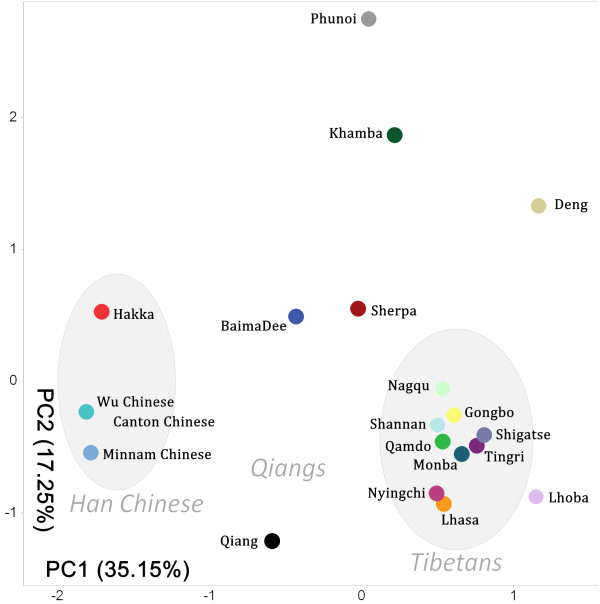
Principal component analysis of the estimated haplotype frequencies of the Sino-Tibetan populations.

To assess the population relationships within the *ADH1B* region, we did principal component analysis based on the estimated haplotype frequencies of the populations (Figure 5). In the first component, the Tibetans and Han Chinese are clearly distinguished, while the Qiang populations are between the Tibetans and Han Chinese. The Deng and Sherpa are obvious outliers from the central Tibetan populationswith the Sherpa closer to the Qiang.

**Figure 6:**
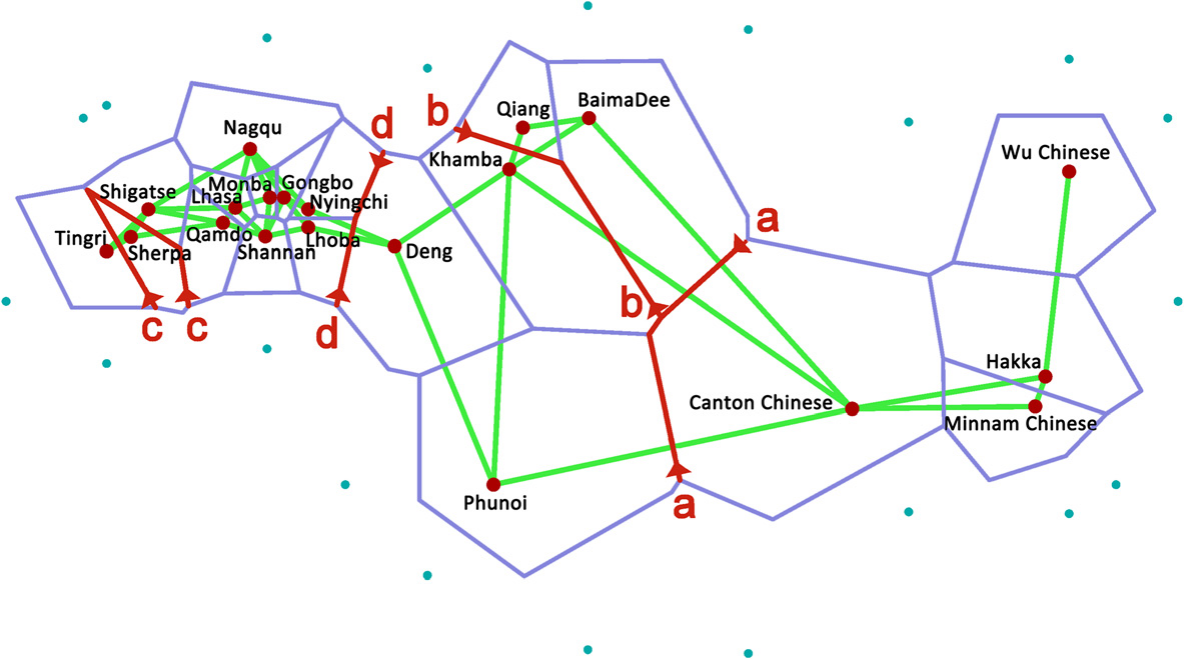
A Delaunay triangulation (green lines) and the genetic barrier (red lines) computed on aFst distance matrix between populations.

We also calculated the population pair-wise Fst values and used the Barrier 2.2 software to identify genetic barriers (Figure 6). The first barrier, a, between Han Chinese and the Tibeto-Burman populations, has the highest distance value (Phunoi 0.238, Khamba 0.234, Baima Dee 0.088). Barrier b appears between the Qiangs and the other Tibeto-Burman populations (Qiang-Khamba 0.110, Baima Dee-Khamba 0.056). Then, Sherpa and Deng are also excluded from the Tibetans by barriers c and d. This result matches that of the principal component analysis, showing that the Qiangs are placed genetically between the Tibetans and Han Chinese.

## Discussion

### *ADH1B* H7 arose in the common ancestors of Sino-Tibetan populations

In East Asia, the *ADH1B* gene is one of the genes whose diversity is correlated with ethnic classifications. Frequencies of *ADH1B*haplogroups are very different among different ethnic groups (linguistic families) [12]. Compared to the high frequency of H7 in Han and Hmong Chinese, the frequency of H7 is rather low in the Tibetans (approximately 12%) and other populations (approximately 5%) in Tibet. However, the haplotype diversity of H7 reaches the highest value in the Tibetans, indicating a long history of this haplogroup in Tibet. Network analysis showed that most H7 haplotypes in the Tibetans have quite different flanking sequences from those that occur in Han Chinese. Thus, H7 has diverged in these two populations for a long time, and the origin of H7 might not be in either of the populations. The Tibetans and Han Chinese both speak Sino-Tibetan languages. Genetic and linguistic studies indicate that these two ethnic groups originated in the common ancestors in the upper reaches of the Yellow River about 6,000 years ago [20,23]. *ADH1B* H7 might have come from the common ancestors of the Sino-Tibetan populations. Historical records say that the Tibetans came from the ancient Qiang people [47], which is the original population of Sino-Tibetan people. In our present Qiang sample, the H7 haplotype diversity is not the highest, but the average nucleic diversity is the highest, indicating a great age of H7 in the Qiang. However, our Qiang sample is from only one of the various Qiang populations [48-50]. Other Qiang populations should be included in future investigation to provide a better, more detailed evolutionary history of the *ADH1B* gene in East Asia.

### Why is there no signal of selection on*ADH1B* H7 in Tibet?

Signals of selection on *ADH1B* H7 are strong in Han Chinese, Japanese, Koreans, and Hmong. In *ADH1B* H7, both alleles of the non-synonymous rs1229984 and regulatory region rs3811801 are derived. We did not find samples with only the derived allele of rs3811801 in the previous studies [12], and therefore, we cannot be sure if the derived allele of rs1229984 is sufficient to explain the selection. In this study, we found a new haplotype, H7b with only the derived allele of rs3811801 in the Tibetans, but we are not yet sure whether both derived alleles are necessary for selection as the diversity of H7b is too high.

*ADH1B* H7 was derived from *ADH1B* H6 [14]. In those East Asian populations lacking selection signals at *ADH1B*, the frequencies of H6 are all much higher than the derived haplogroup H7. The frequency of H7 is much higher than H6 and appears to have increased rapidly as the result of selection in Han Chinese, Japanese, and so on [11,12]. In Tibetans, the frequency of H7 is also much higher than that of H6, which could also suggest positive selection. However, the LRH test revealed only a weak, non-significant signal of selection in the Tibetans. That may also explain why the frequency of H7 in Tibetans is not as high as in Han Chinese.

The selection of the 48His variant of *ADH1B* in East Asia appears related to agriculture, most likely to rice domestication [13]. In Tibet, the major lifestyle is not crop farming but stock farming [51], and the few crops in Tibet are highland barley and millet [52], not rice. This might be the reason selection on *ADH1B* is not detectable in Tibet. Furthermore, the crops can be better stored on the cold highland than on the warm plain. While no definitive explanation of what characteristic was the basis of selection, these data are consistent with one hypothesis related to toxins from decomposition during storage [53]: fewer toxins would be generated during crop storage in Tibet, and therefore, the selective force on the *ADH1B* gene would be small to absent.

## Conclusions

The diversification of *ADH1B* in the Sino-Tibetan populations has a long history. Haplogroup H7 of *ADH1B* originated in the ancestor of Sino-Tibetan populations and flowed to the Tibetans very early. However, as the Tibetans have a lifestyle less dependent on crops, selection has not had significant effects, and H7 has not risen to a high frequency, whereas the diversity of the haplogroup has accumulated to a very high level.

## Competing interests

The authors declare that they have no competing interests.

## Authors’ contributions

HL and LK designed the study. HL, YL, KKK, and JRK wrote the manuscript. LK, KH, and FC carried out the field investigation and sample collection. YL, KH, CW, and XS performed the experiments. HL, YL, LK, and CW analyzed the data. All authors read and approved the final manuscript.

## Supplementary Material

Additional file 1 **Table S1.** Haplogroup diversity of Class I *ADH* and *ADH7* among Tibetan populations. Click here for file

Additional file 2 **Figure S1.** HIS tests for the *ADH1B* region of the Tibetan populations. Click here for file
