# Supplementary figures and images for: High diversity and no significant selection signal of human ADH1B gene in Tibet
Source: Investig Genet. 2012 Nov 23;3:23. doi: 10.1186/2041-2223-3-23 (PMC3528464; doi:10.1186/2041-2223-3-23)

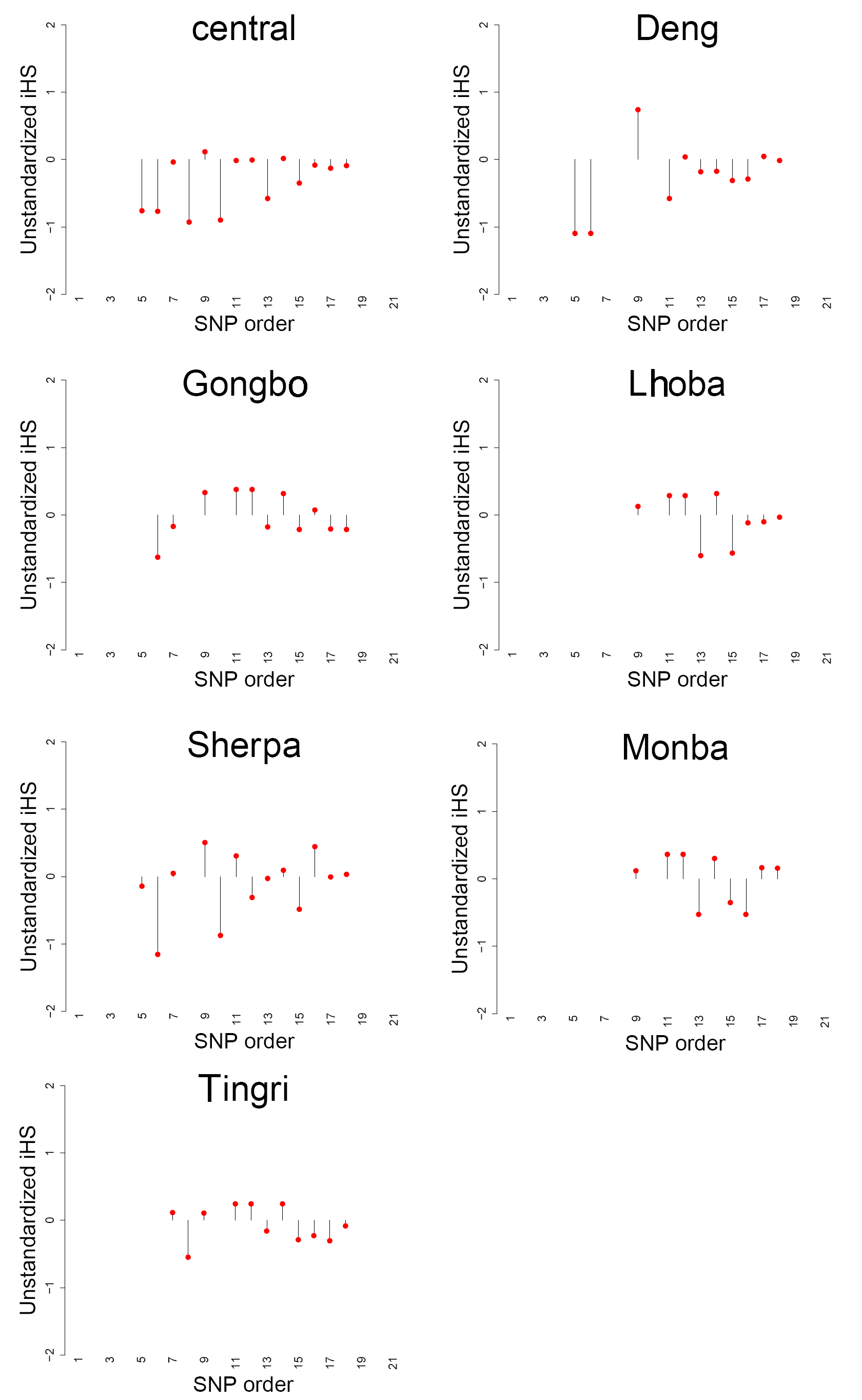

Supplement: Additional file 2 — Figure S1. HIS tests for the ADH1B region of the Tibetan populations. [file 2041-2223-3-23-S2.tiff]
